# Targeting-intratumoral-lactic-acidosis transcatheter-arterial-chemoembolization for non-islet cell tumor hypoglycemia secondary to a liver metastatic solitary fibrous tumor: A case report and literature review

**DOI:** 10.3389/fendo.2022.955687

**Published:** 2022-08-11

**Authors:** Kai Jin, Shan Zhong, Liya Lin, Jianjun Wu, Yuqi Wang, Weijuan Cui, Wei Gu, Ming Chao, Xiaoxiao Song

**Affiliations:** ^1^ Department of Radiology, The Second Affiliated Hospital School of Medicine, Zhejiang University School of Medicine, Hangzhou, China; ^2^ Department of Endocrine and Metabolic Diseases, The Second Affiliated Hospital, Zhejiang University School of Medicine, Hangzhou, China; ^3^ Clinical Research Center of the Second Affiliated Hospital, Zhejiang University School of Medicine, Hangzhou, China; ^4^ Cancer Institute of the Second Affiliated Hospital, Zhejiang University School of Medicine, Hangzhou, China; ^5^ Department of Endocrine and Metabolic Diseases , The First People’s Hospital of Linping District, Hangzhou, China

**Keywords:** TILA-TACE, solitary fibrous tumor, Doege–Potter syndrome, non-islet cell tumor, hypoglycemia

## Abstract

Doege–Potter syndrome is a rare paraneoplastic syndrome characterized by non-islet cell tumor hypoglycemia secondary to a solitary fibrous tumor. Doege–Potter syndrome always presents with recurrent fasting hypoglycemia, which can occasionally be life-threatening. The best choice of treatment for Doege–Potter syndrome and solitary fibrous tumor is complete resection. However, when it is unfeasible, local-regional treatment can be used as a palliative therapy. Herein, we report a case of a 46-year-old man with Doege–Potter syndrome that occurred secondary to the liver and pancreatic metastatic solitary fibrous tumors. After he received six rounds of targeting-intratumoral-lactic-acidosis transcatheter-arterial-chemoembolization (TILA-TACE) treatment in our hospital, his hypoglycemia was clinically cured, and the liver metastatic tumor was well controlled. We suggest that TILA-TACE can be considered when curative resection is unfeasible for metastatic liver solitary fibrous tumors to help a patient obtain further surgery opportunities.

## Introduction

Solitary fibrous tumors (SFTs) are rare neoplasms that originate from mesenchymal spindle cells. They were first reported by Wagner in 1870, and their histopathological report was subsequently described by Klemperer and Rabin in 1931 (1). In 2013, based on the World Health Organization (WHO) classification of tumors of soft tissue and bone, SFTs were considered benign tumors with potential for malignant transformation (2). Most SFTs are inert, without local or distant recurrence. However, the 10-year disease-specific survival rate for pleural and extra-pleural SFT is about 73%–100%, and the 10-year recurrence rate is 10%–25% (3–8). Meningeal hemangiopericytoma in the central nervous system and SFT were classified as the same tumor by WHO. On the other hand, hemangiopericytoma/SFT is more aggressive; local recurrence is frequent and rapid, with the possibility of meningeal spread and early distant metastasis to the bone. According to the WHO classification, though most of SFTs are considered benign, some SFTs can be considered malignant with the following histopathologic characteristics: cytological atypia, hypercellularity, tumor necrosis, high mitotic rate (>4 per high-power field (HPF)), and/or infiltrative margins (9). At present, it is not recommended to use “benign” or “malignant” to evaluate the prognosis of patients; it is more applicable to apply the risk of recurrence/metastasis for SFTs. Patients with the following conditions are more likely to have tumor recurrence/metastasis: incomplete surgical resection, metastatic disease at the time of visiting, tumor greater than 10 cm, high mitosis rate (>4 mitotic images/10 HPF), and tumor necrosis (4, 10–14). Ki-67 greater than 5% is also considered a marker (15). The clinical manifestations of SFTs are usually nonspecific. Hypoglycemia occurs in approximately 5% of SFT cases, in a rare and challenging paraneoplastic syndrome known as the Doege–Potter syndrome (DPS), characterized as non-islet cell tumor hypoglycemia (NICTH) (16, 17). DPS usually manifests as a rare, refractory, and severe hypoglycemia.

For both SFTs and DPS, the best treatment is complete resection of the tumor. However, when curative resection is not feasible, short-term therapy, including continuous intravenous infusion of glucose and medical therapy, is beneficial. Other treatments include adjuvant chemotherapy and radiotherapy (18, 19). It is reported that pazopanib can effectively control the progression of the tumor and can be considered a first-line treatment for some advanced typical SFTs (20–23). Other drugs, like temozolomide and bevacizumab, could also play a role. However, more study is needed to verify the effects of these drugs (24). For primary and metastatic liver SFTs, local-regional treatments such as transcatheter arterial chemoembolization (TACE) have been reported (19, 25). Zhong et al. have recently reported a case of hepatic SFT in a patient who was treated with curative *ex situ* hepatectomy and liver autotransplantation (26). All of these approaches can play a part in treating hepatic SFTs.

Herein, we report the case of a patient with DPS caused by multiple metastatic SFTs in the liver and pancreas. Due to multiple liver metastases and serious hyperglycemia, he was not a suitable candidate for direct surgery. He had previously been treated with diet therapy, continuous infusion of glucose, low-dose glucocorticoid treatment, and sorafenib tosylate, as well as four cycles of conventional TACE (cTACE) in other hospitals, yet experienced little significant improvement. Fortunately, in our hospital, his hypoglycemia was clinically controlled after six cycles of targeting-intratumoral-lactic-acidosis (TILA)-TACE, and his metastatic liver tumors became almost necrotic after eight cycles of TILA-TACE treatment, which was first reported by our team (27). The patient then underwent a successful combined operation of the pancreatic body and tail resection, splenectomy, and left lateral lobe hepatectomy after his hypoglycemia was clinically cured and the further progression of SFTs was controlled.

In this case, our patient benefited from TILA-TACE rather than the original cTACE treatment. Hence, we suggest that for patients with primary and metastatic liver SFTs and who are not eligible for curative surgery, TILA-TACE may be a successful therapeutic alternative.

## Case report

**Figure 1:**
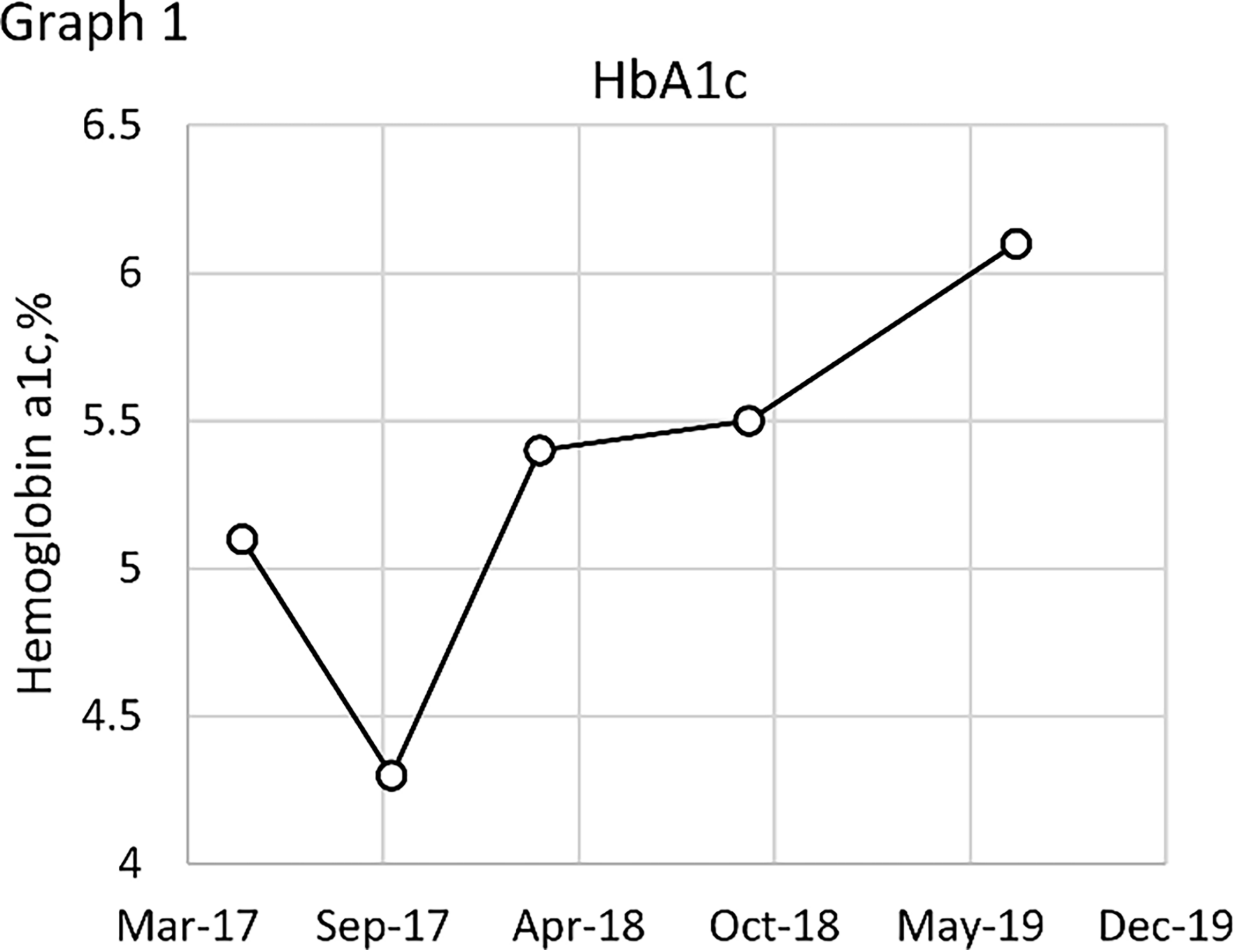
Contrast-enhanced magnetic resonance imaging (MRI) of the liver before TILA-TACE. Contrast-enhanced MRI of the liver showed at least six large masses in the liver, the largest one of which was approximately 14 cm in diameter. All of the masses demonstrated a slightly low mixed signal on T1WI **(A)** and a slightly high mixed signal on T2WI **(B)**; after contrast, all these masses were clearly enhanced **(C**, **D)**. In addition, a mass located in the tail of the pancreas that measured 3.6 cm in diameter was observed (arrow), with short T1 **(E)** and long T2 **(F)** signals on MRI, and the mass was evidently enhanced after contrast **(G**, **H)**.

A 46-year-old Chinese man visited our hospital for recurrent paroxysmal unconsciousness occurring for 10 months (from January 2017). At first, his hypoglycemia usually occurred in the morning, and later it was irregular, accompanied by sweating, weakness, and occasional urinary incontinence, and these symptoms disappeared after eating. He had no history of diabetes but had a history of drinking for 20 years and had been sober for 1 year before the onset of hypoglycemia. The results of laboratory test were as follows: serum blood glucose at 1.26 mmol/L (normal range: 3.89–6.11 mmol/L), serum insulin level at <3.48 pmol/L (normal range: 17.8–173.0 pmol/L), C-peptide at 0.03 nmol/L (normal range: 0.27–1.28 nmol/L), and insulin-like growth factors IGF-1 at < 25.0 ng/ml (normal range: 94.0–252 ng/ml). Glutamate acid decarboxylase antibodies, anti-insulin autoantibodies, and anti-islet cell antibodies were negative. Positron emission tomography showed scattered low-density lesions in the liver and an enlarged lymph node behind the pancreas with increased glucose uptake. Liver contrast-enhanced magnetic resonance imaging (MRI) was performed in May 2017 and revealed pancreatic and multiple liver masses, showing at least six large masses (diameter >5 cm) and multiple small metastases in the liver, the largest one of which was approximately 14 cm in diameter. All of the masses demonstrated a slightly high mixed signal on T2-weighted imaging and diffusion-weighted imaging and a slightly low mixed signal on T1-weighted imaging. In contrast, all these masses showed obvious enhancement. In addition, a mass was found located in the tail of the pancreas that measured 3.6 cm in diameter with short T1 and long T2 signals on MRI, and the mass showed evident enhancement after contrast. Neuroendocrine tumors were first considered (**Figure 1**). Later, liver mass biopsy and immunohistochemistry results showed positive staining with antibodies against STAT6 and CD34. Thus, the patient was diagnosed with SFT and DPS, and initially received four cycles of cTACE to reduce the side effects as direct surgery might be life-threatening. However, no improvement was observed, and the patient still had severe hypoglycemia. Previous medical history showed that he had resection of a right fossa pterygopalatine tumor in 2010, and reoperation in 2012 as the tumor recurred; a further pathology report showed a spindle cell tumor diagnosed as a hemangiopericytoma.

**Figure 2:**
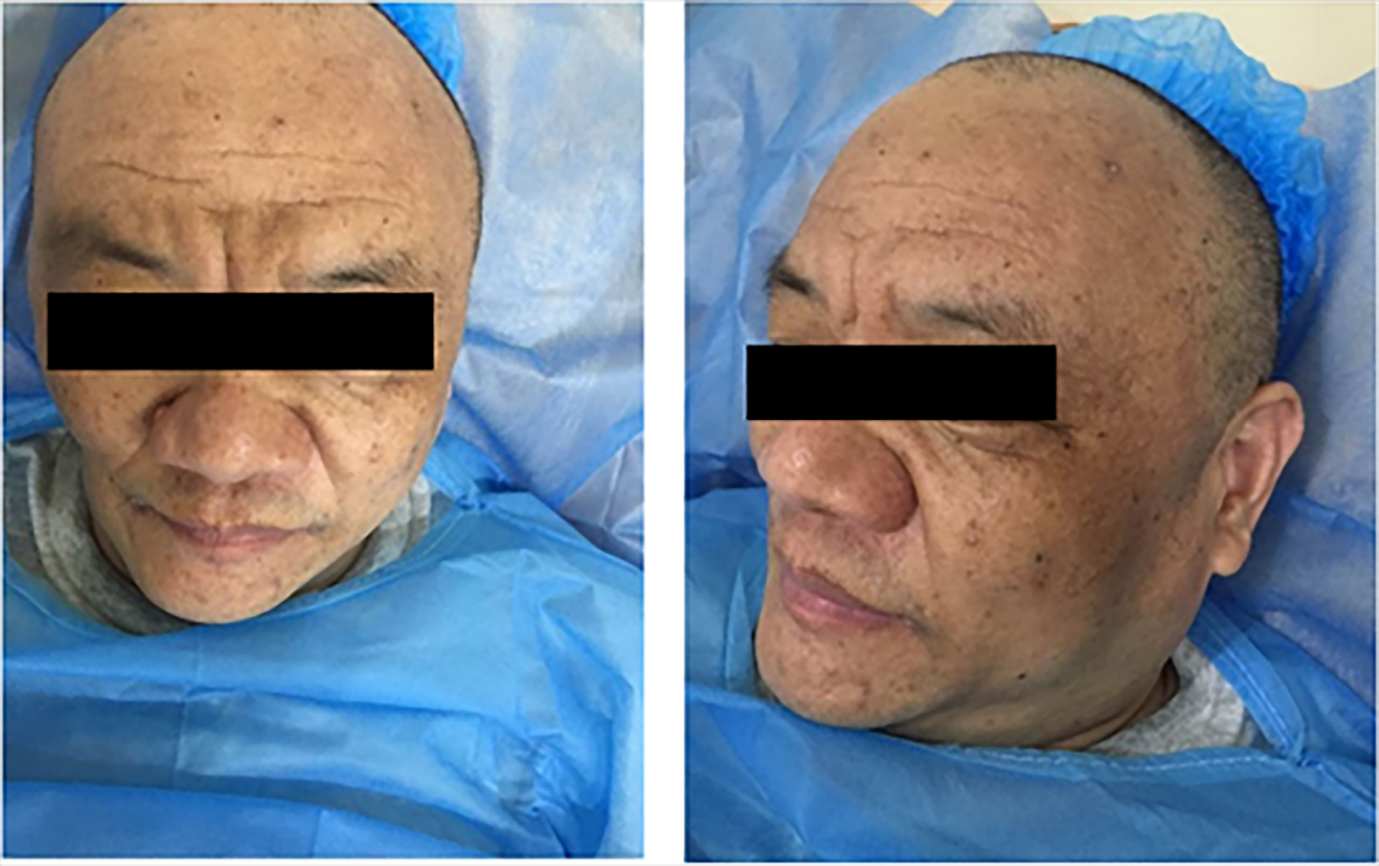
Facial changes of the patient, including acrochordons and rhinophyma.

On physical examination, he was an overweight man with a body mass index of 29.01 kg/m^2^. Facial changes included acrochordons and rhinophyma (**Figure 2**). Abdominal bulging was observed, and abdominal palpation revealed an enlarged liver. No other physical abnormalities were found.

Laboratory data showed fasting hypoglycemia, and the results were as follows: serum blood glucose at 1.25 mmol/L (normal range: 3.89–6.11 mmol/L), serum insulin level at <3.48 pmol/L (normal range: 17.8–173.0 pmol/L), C-peptide at 0.03 nmol/L (normal range: 0.27–1.28 nmol/L), IGF-2 at 1,964.33 ng/ml (normal range: 400–736 ng/ml), IGF-1 at <25.0 ng/ml (normal range: 94.0–252 ng/ml), pro-IGF-2 at 28.79 ng/ml, and IGF-2/IGF-1 >10. Tumor markers such as alpha-fetoprotein (AFP), carbohydrate antigen 19-9 (CA19-9), and carcinoembryonic antigen (CEA) were negative. Routine biochemical indicators such as transaminase were normal in laboratory tests. Thyroid dysfunction, functional islet cell tumors, and adrenal dysfunction were excluded. Head MRI showed no obvious abnormalities except the VR space of the right cerebral peduncle and the lacunar foci in the right semioval area. Enhanced MRI of the nasopharyngeal area showed postoperative changes in the right pterygopalatine fossa tumor. There were multiple nodular abnormal signals in the right temporal fossa that may be a local recurrence. A contrast-enhanced computed tomography (CT) scan of the abdomen showed multiple nodules and masses of varying sizes in the liver, with unclear borders, which were enlarged compared to May 2017. The largest one was located in the eighth segment of the liver, with a diameter of 15.9 cm. The masses were unevenly enhanced in the arterial phase; in the portal phase and the delayed phase, the enhancement of the mass was weakened but still higher than the surrounding liver tissue. A mass in the posterior and lower parts of the pancreas was noted that was unevenly enhanced with a blurred border.

Based on the aforementioned evidence, the patient was diagnosed with metastatic liver and pancreatic SFT, along with the manifestation of DPS. We speculated that the right fossa pterygopalatine tumor was the primary SFT based on the WHO classification of hemangiopericytoma/SFT. Intravenous boluses of 10% dextrose were administered to correct his hypoglycemia, and his blood glucose was monitored through capillary tests and a blood glucose instantaneous sensor. There was a decrease in the frequency of the episodes of unconsciousness, but they occurred irregularly at least once a day.

**Graph 1:**
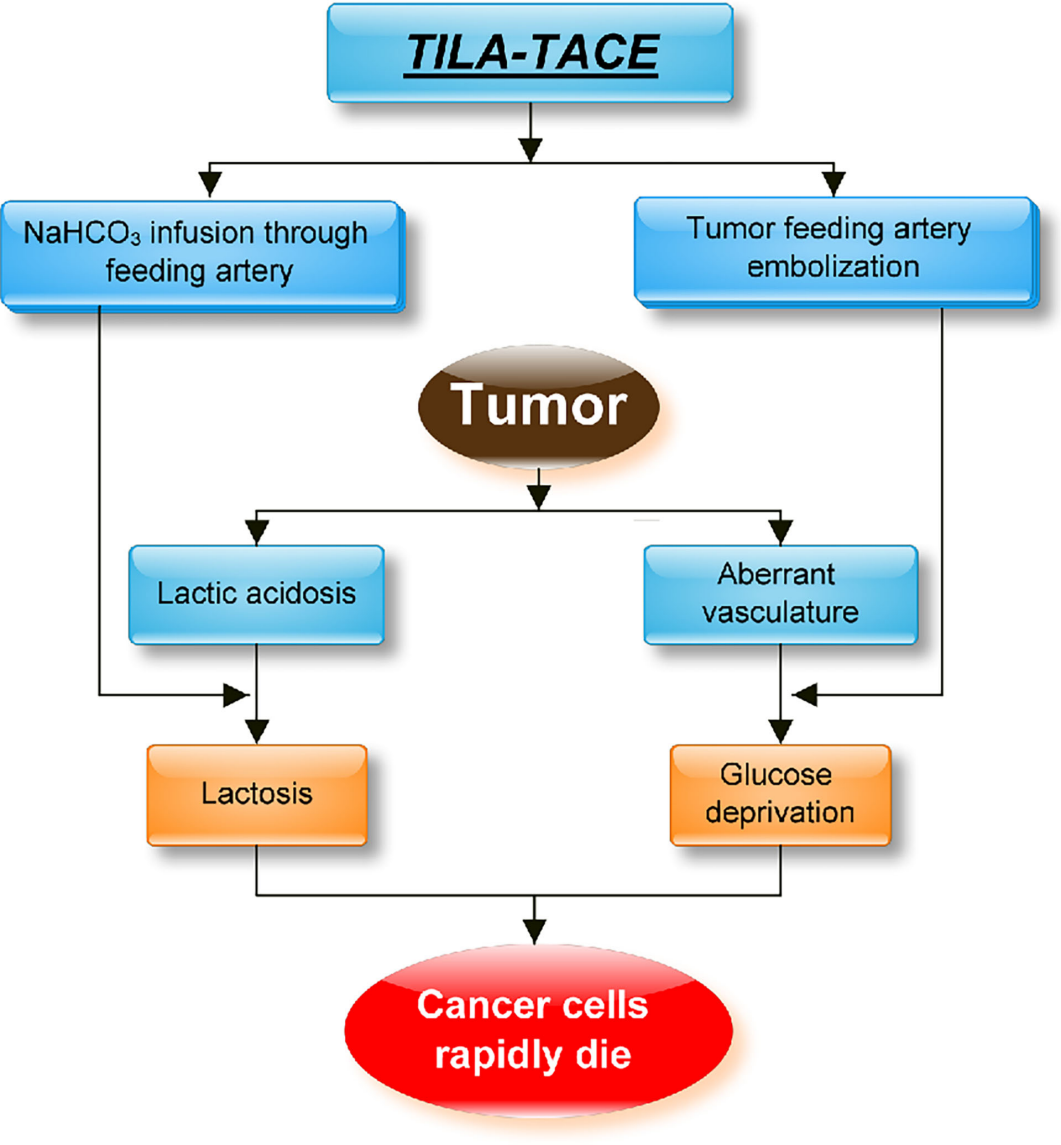
HbA1c during hospitalization, before and after TILA-TACE treatment.

**Figure 3:**
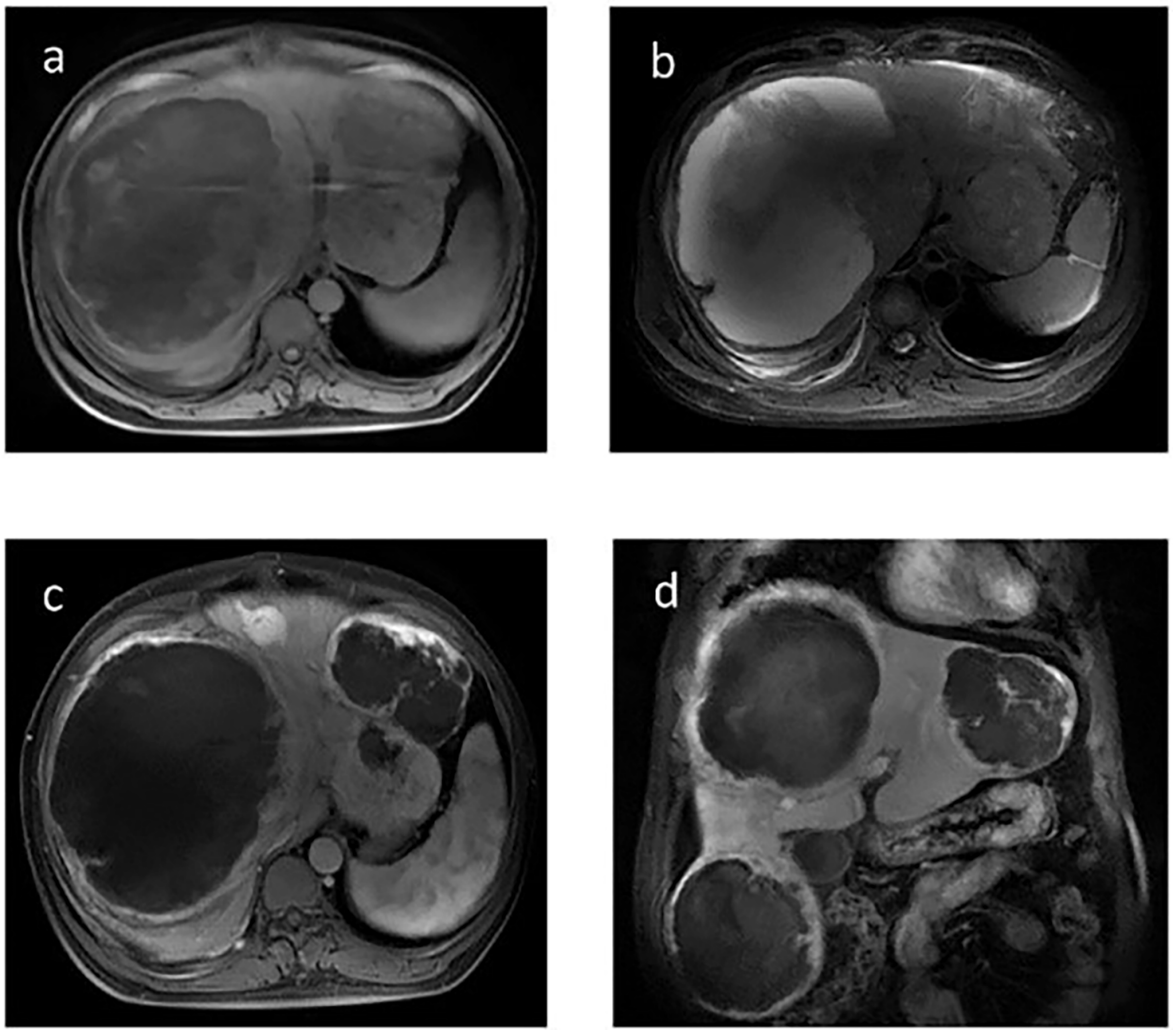
Contrast-enhanced MRI of the liver after the third TILA-TACE cycle showed that all of the masses demonstrated slightly low mixed signal on T1WI **(A)** and high mixed signal on T2WI **(B)**. After contrast, most of the masses were almost necrosed **(C**, **D)**.

The effects of diet and drugs were poor, cTACE did not work, and surgery was unavailable; thus, after a multidisciplinary discussion, he was treated with TILA-TACE therapy. Laboratory tests after the first TILA-TACE showed that big-IGF-2 and IGF-2 had obviously decreased, 18.05 and 922.33 ng/ml, respectively, and the serum blood glucose level had also significantly improved. However, hypoglycemia recurred after 3 days at a frequency and severity similar to those before TILA-TACE. One month later, after the second TILA-TACE cycle, the recurrence-free time had extended to 1 week. When the third TILA-TACE cycle was completed, the symptoms and the blood glucose level significantly improved, and no drug treatment was required. Hemoglobin A1c (HbA1c) during hospitalization before and after TILA-TACE treatment is shown in **Graph 1**. Insulin, C-peptide, IGF-1, HbA1c, and glucose levels during treatment are shown in **Table 1**. Contrast-enhanced MRI of the liver performed after the third cycle of TILA-TACE showed that almost all of the large liver lesions had complete necrosis (**Figure 3**). After six cycles of TILA-TACE, the patient’s blood glucose was basically back to normal; no extra meals were required.

**Table 1:** The patient’s insulin, C-peptide, IGF-1, and hemoglobin A1c throughout the whole treatment.

Date	Glucose (mmol/L)	Insulin (pmol/L)	C-peptide (nmol/L)	IGF-1 (ng/ml)	Hemoglobin A1c (%)	
2017-05	1.26	<3.48	0.03	<25.0	5.1	
2017-10	1.25	<3.48	0.05	<25.0	4.3	After four times of cTACE
2017-11^a^	5.00	NA	NA	NA	NA	
2017-12^a^	4.80	NA	NA	NA	NA	
2018-02^a^	4.90	NA	NA	NA	NA	
2018-03	3.66	0.6	0.07	<25.0	5.4	1 week after the third round of TILA-TACE
2018-10	3.70	4.4	0.05	<25.0	5.5	2 months after the sixth round of TILA-TACE
2018-11	4.31	9.42	0.12	<25.1	NA	7 days after the surgery
2019-05^a^	6.81	NA	NA	NA	NA	10 days after the last (eighth) round of TILA-TACE
					6.1 (2019‐07)	

a TILA-TACE operation time.

**Figure 4:**
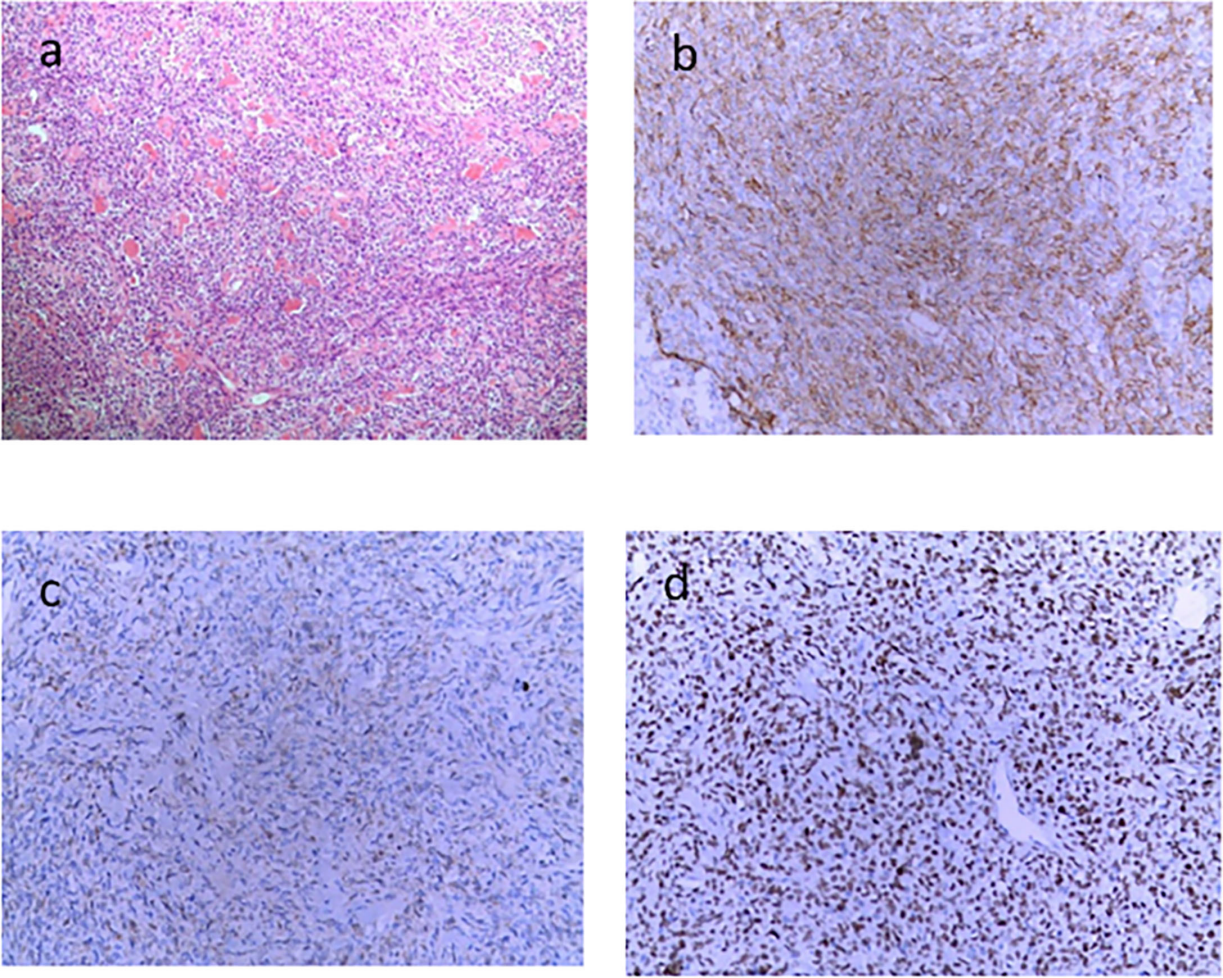
Microscopic examination of the pancreatic lesion (6.5 × 5 cm in size) demonstrated the presence of spindle-shaped cells, abundant tumor cells with atypia, approximately four to five mitotic figures/10 HPFs, and local infarction, in accordance with the World Health Organization grade III. **(A)** HE staining (×100). **(B)** CD34 immunohistochemical stain (×200). **(C)** BCL2 immunohistochemical stain (×200). **(D)** STAT-6 immunohistochemical stain (×200).

After the glucose level had stabilized, the patient underwent a combined operation of the pancreatic body and tail resection, splenectomy, and left lateral lobe hepatectomy after six cycles of TILA-TACE treatment. Due to multiple liver and pancreatic metastases, the surgeon did not suggest complete resection of the tumor. A pathological examination of the pancreas revealed a spindle cell tumor with an abundance of heterotypic cells. Microscopic examination with hematoxylin–eosin staining showed that the tumor had a high proliferation rate of four to five mitotic figures per 10 HPF, and the margin was negative. Immunohistochemistry findings revealed positive staining with antibodies against CD34, Bcl-2, STAT6, and CD31, and Ki-67 was 10% (**Figure 4**). After surgery, his blood glucose was basically back to normal (**Table 1**), and no extra meals were required. Unfortunately, we determined that the residual tumors had progressed, so two more cycles of TILA-TACE were performed after surgery.

**Figure 5:**
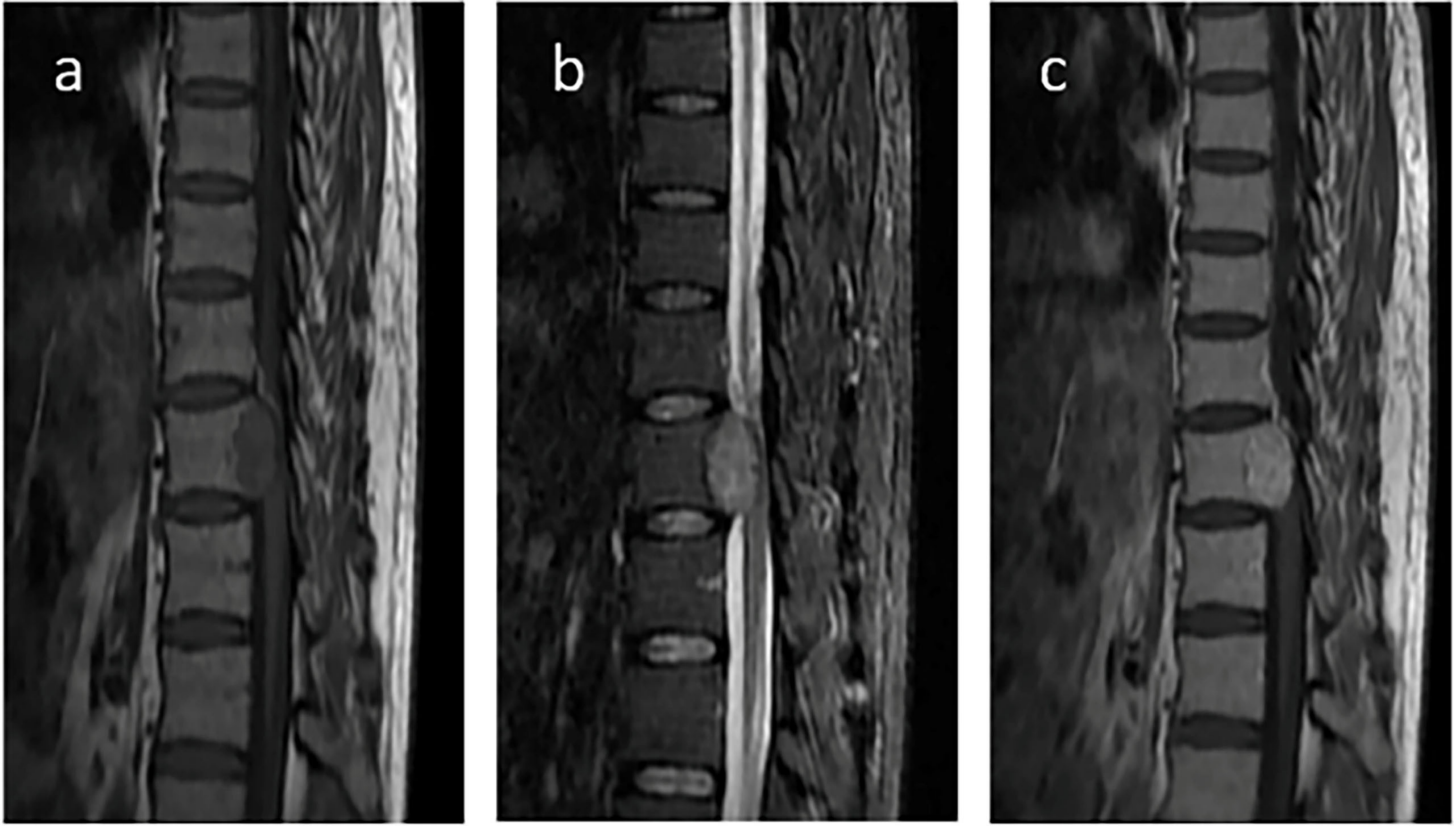
Contrast-enhanced magnetic resonance imaging (MRI) of the thoracic spine. Contrast-enhanced MRI of the thoracic spine confirmed thoracic spine metastasis and one metastatic nodule projected into the spinal canal and compressed the spinal cord. **(A)** T1WI; **(B)** T2WI; **(C)** T1WI postcontrast.

**Figure 6:**
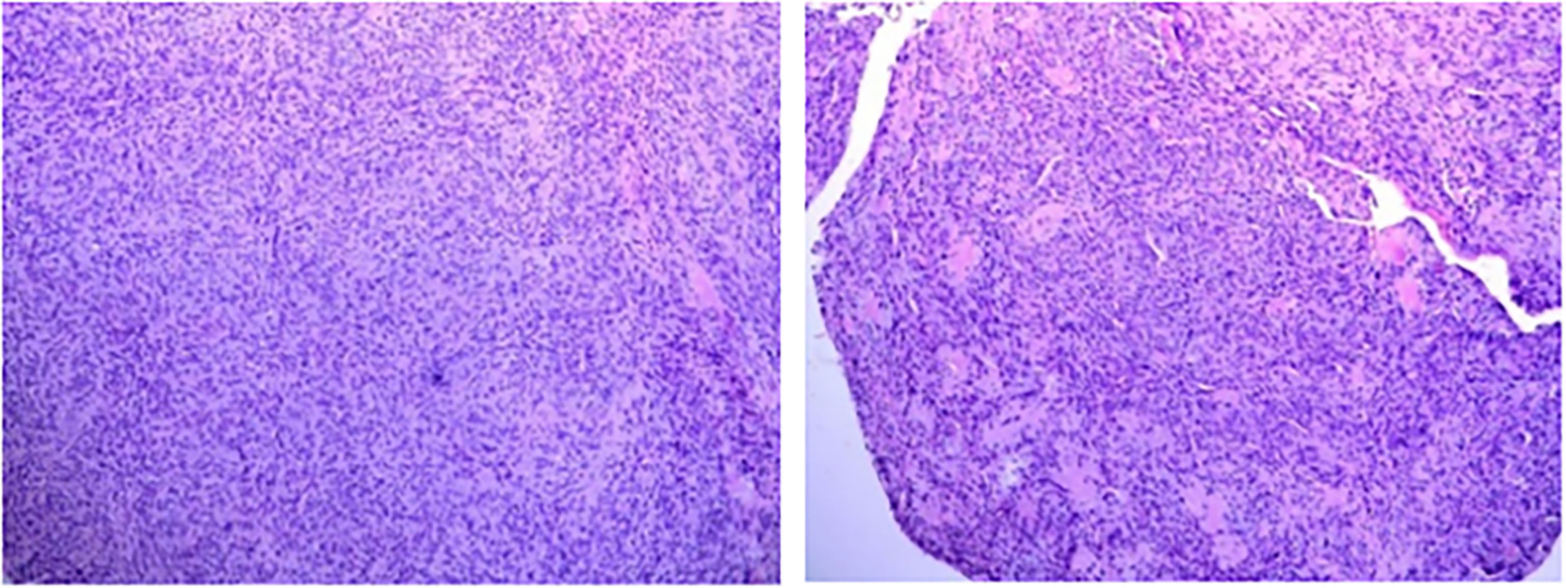
Microscopic examination of the thoracic spine metastatic tumor with HE staining (×100).

Eight months after surgery, the patient was readmitted to our hospital because of instability while walking and left lower limb weakness. Contrast-enhanced MRI of the thoracic spine confirmed thoracic spine metastasis, and one metastatic nodule had projected into the spinal canal, compressing the spinal cord (**Figure 5**). A review of his previous imaging revealed that a metastatic thoracic spine tumor had been present in November 2017. After an uneventful thoracic spine tumor resection, he recovered well with no paraplegic symptoms. Histopathology also showed a spindle cell tumor (**Figure 6**).

Since 2017, the patient has received a total of eight cycles of TILA-TACE and has undergone a combined operation of the pancreatic body and tail resection, splenectomy, and left extrahepatic tumor resection and thoracic spine tumor resection in our hospital. He last visited our hospital for an evaluation in May 2020, and, according to his examination results, the disease was thought to be temporarily stable and his hypoglycemia clinically cured. There is no doubt that TILA-TACE is effective for metastatic liver SFTs and DPS. However, the subsequent treatment of the patient remains an open question because of multiple metastases. Due to economic and personal reasons, the patient did not want to receive further drug or surgical treatment for SFTs after March 2020. The patient died following tumor progression 7 months ago, before the submission of this paper. He had maintained stable blood glucose before his death, with no obvious hypoglycemia events occurring according to his family’s description.

## Discussion

DPS is mediated by several mechanisms. The first mechanism is the consumption of glucose by a large tumor (28). However, some studies have reported that hypoglycemia did not occur again when the tumor recurred and regrew to its former size, indicating that glucose consumption by the tumor may not be the main cause of hypoglycemia (28). Second, abnormalities in the *EGR-*IGF system have been reported to lead to hypoglycemia (28). It is generally believed that the enhanced insulin-like effect is caused by oversecretion of big IGF-2 (29). Moreover, *IGF2* is an *EGR* target gene and is regulated by the chimeric transcription factor *NAB2-STAT6*, leading to abnormalities. NAB2-STAT6 gene fusion induced proliferation in cultured cells and activated the expression of EGR-responsive genes (30). The fusion of *NAB2* and *STAT6* produced the *NAB2-STAT6* chimeric transcription factor, which is located in the nucleus, where it is currently believed to drive tumorigenesis by constitutively activating *NAB2* target genes (30). The *NAB2-STAT6* fusion gene, as a unique molecular feature of SFT, appears in up to 100% of cases and has not yet been detected in other tumors (31). RT-PCR detection can be used to identify the *NAB2-STAT6* fusion gene, but due to the diversity of fusion types, its sensitivity is much lower than that of STAT6. Therefore, STAT6 detection is more widely used in clinical settings (32–34).

The IGF system consists of two ligands, IGF-1 and IGF-2, as well as their two receptors. Normally, approximately 70%–80% of IGFs bind to insulin-like growth factor binding protein (IGFBP)-3 in serum, whereas residual IGFs bind to other IGFBPs, leaving less than 1% of IGFs free (28). This mechanism effectively protects IGFs from degradation and avoids hypoglycemia by limiting their binding to receptors (28, 35). Bertherat et al. studied the specific expression of the *IGF-2* gene in NICTH/DPS caused by pleural fibrosarcoma (36). They observed a loss of imprinting of parent alleles, which resulted in an excess expression of the *IGF-2* gene and an increase in incompletely processed IGF-2, termed big-IGF-2 or pro-IGF-2. Moreover, tumor cells did not seem to have enough enzymes to act on pro-IGF-2; thus, the excess pro-IGF-2 competes with IGF-1 and IGF-2 in binding to IGFBP to form a 40–50–kDa binary complex. In that case, the process results in an excess of free IGF-2 (37) and IGF-1 in the plasma (38, 39). The increased IGF-1 causes a negative feedback, leading to a decrease in the upstream GH secretion, followed by a decline in IGF-1 and IGFBP-3 levels. IGF-2, on the other hand, will not be reduced since its secretion is not regulated by feedback because it is automatic and paracrine (20). This leads to an increased ratio of serum IGF-2/IGF-1 and an inversely proportional relationship between IGFBP-3 and IGF-2. Meanwhile, the binary complex and free IGF-2 pass through capillary membranes relatively easily and bind to insulin receptors, subsequently causing hypoglycemia (35). In DPS, as pro-IGF-2 tests have not been commercialized, the IGF-2:IGF-1 ratio is considered to be a surrogate marker for pro-IGF-2. DPS is diagnosed when the IGF-2:IGF-1 ratio is greater than 10 (40).

The best treatment for SFTs is complete resection of the tumor. Nevertheless, when curative resection is not available, metastasectomy, chemotherapy, and radiotherapy may be beneficial (18, 19). A retrospective work (*n* = 64) suggested that surgical resection of localized SFT can result in a 10-year overall survival (OS) of 58% when compared to that of metastatic SFT, which is 11% (21). Although there are various chemotherapies, no standard regimen has been recommended for metastatic SFTs. Several case reports and small sample studies have reported that pazopanib effectively controlled the progression of the tumor in metastatic SFTs, and the best median progression-free survival rate was 6.2 months (22). When it comes to metastatic liver SFTs, TACE may be a good alternative therapy. Velayati et al. summarized the safety and efficacy of the treatment of TACE (19). However, TACE may not always be effective. El-Khouli et al. reported the first use of cTACE: after three cycles of TACE treatments, no significant reduction in tumor size was observed (41). This is a condition similar to our patient. The reason for the low response of cTACE in metastatic liver SFTs needs further studies.

With regard to DPS, the therapies include the following: correct hypoglycemia immediately and treat the potential tumor or prevent recurrent episodes of hypoglycemia when the tumor cannot be controlled. There are several ways to correct hypoglycemia, but the best treatment is still surgery. Administrating quick-acting carbohydrates (such as glucose tablets, sugared fruit juices, or hard candy), intravenous glucose infusion, or injection of glucagon are good methods to correct hypoglycemia immediately (42). When the tumor secretes IGF, complete removal of the tumor can cure hypoglycemia (43). If surgery is not available, the methods mentioned above and glucocorticoid therapy can be considered alternatives. In spite of that, the efficacy is clear but not entirely curative. Several case reports have shown that TACE is a good choice for patients with liver SFT and DPS (25, 44). However, in these cases, there were no multiple metastases and some patients need multimodal treatment to control hypoglycemia after cTACE treatment (43). We speculated that cTACE treatment still has some limitations on both SFTs and DPS.

**Figure 7:**
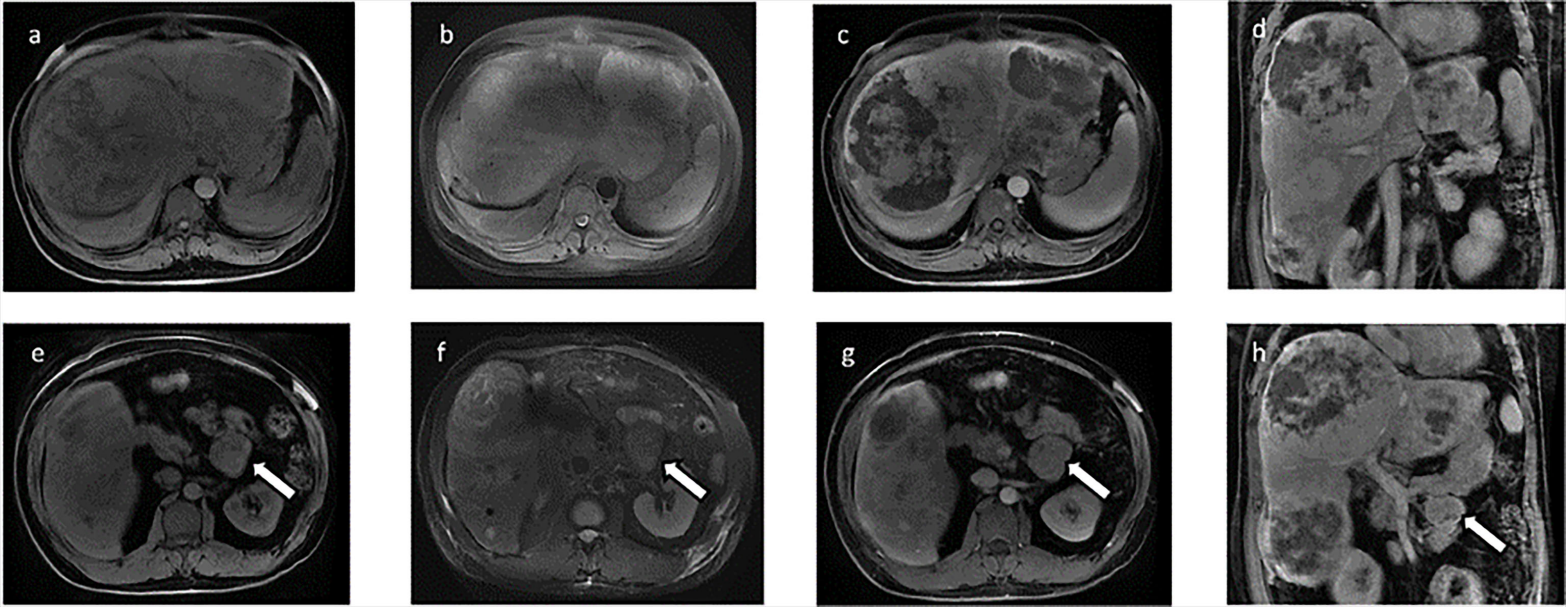
The hypothetical approach of TILA-TACE to treating large liver metastatic solitary fibrous tumors by targeting intratumoral lactic acidosis. cTACE embolizes tumor-feeding arteries that block glucose supply but also create a hypoxia condition and trap lactic acidosis. Intratumoral lactic acidosis rescues cancer cells from glucose deprivation. Hypoxia-enhanced angiogenesis could also significantly contribute to tumor survival. On the contrary, TILA-TACE is designed for neutralizing lactic acidosis by bicarbonate, which rapidly kills cancer cells before revascularization and thus significantly improves the therapeutic efficiency.

TILA-TACE is a powerful method to treat both SFTs and DPS. It can effectively induce tumor cell necrosis, which is more effective than that induced by cTACE. The mechanism of cTACE is by embolizing blood vessels and then creating a microenvironment with a low concentration of glucose, which affects energy metabolism and causes tumor cell death. However, in the condition of our patient, he had no improvement in blood glucose and tumor prognosis after four cycles of cTACE. The possible reasons were as follows: firstly, we know cTACE causes hypoxia and glucose deprivation, but hypoxia can simultaneously induce transcriptional activation of the *IGF-2* gene, leading to increased production of pro-IGF-2, which compromises the effect of tumor cell death (28); secondly, tumor cells can survive with the help of proton and lactate, which may lead to treatment failure (27). cTACE blocks tumor-feeding arteries; the amount of glucose in the embolized tumor is limited, and the low oxygen level speeds up glycolysis and glutaminolysis. As a result, lactate and proton accumulation create a chemical environment called lactic acidosis (high lactate concentration with acidic pH). When glucose is used up, lactate and protons accumulated together can rescue cancer cells from glucose deprivation-induced death. TILA-TACE, on the other hand, using bicarbonate to neutralize tumor bed, can convert intratumoral lactic acidosis to lactosis, which can effectively prevent tumor cells from using glucose and accelerate cell necrosis. This demonstrates a superior activity in the local control of large tumors (45). In our previous nonrandomized study, the 1-, 2-, and 3-year survival rates in patients with large hepatocarcinoma treated with cTACE were lower than those treated with TILA-TACE. The median survival of the former was 14 months, and the median survival time was 41 months for TILA-TACE (*p* < 0.05) (27). The hypothetical therapeutic mechanism of the novel treatment TILA-TACE is presented as a flowchart (**Figure 7**).

Considering the case of our patient, the primary lesion is likely to be intracranial SFT. The patient’s overall survival was about 10 years, with about 40 months after the development of multiple metastatic SFTs. He had severe hypoglycemia caused by metastatic SFTs in the liver and pancreas. Laboratory examinations revealed that fasting hypoglycemia, GH, and IGF-1 levels were lower than the measurable values, along with a low fasting C-peptide level. Additionally, levels of IGF-2, pro-IGF-2, and the molar ratio of IGF-2 to IGF-1 were increased. After cTACE treatment, the symptoms and laboratory tests showed no improvement, and abdominal CT even indicated an enlarged liver tumor. However, after TILA-TACE treatment, most of the metastatic liver SFTs had completely undergone necrosis and the serum levels of pro-IGF-2 and IGF-2 had decreased. Thereafter, his hypoglycemia was significantly improved, and, after tumor partial resection, his blood glucose had improved to normal levels. No other extra meals were required. Overall, TILA-TACE played a vital role in the treatment of our patient: it cured hypoglycemia and provided our patient with surgical opportunities for SFT metastases.

Therefore, it is reasonable to believe that the main cause of DPS in our patient was the excess of IGF-2 and pro-IGF-2. TILA-TACE is not only an effective measure for the treatment of metastatic SFTs but is also applicable to patients with DPS. TALI-TACE can effectively reduce tumor size, control tumor progression, provide patients with surgery opportunities and prolong survival in metastatic SFTs. Meanwhile, based on the good effect of TALI-TACE on the rapidly growing giant liver tumor, we also speculate that TALI-TACE is suitable for patients with primary SFTs in the liver. Overall, the effect of intratumoral lactic acidosis on tumor cells in a combination of hypoxia-enhanced revascularization significantly contributes to the cTACE therapeutic bottleneck (46). Whereas, destroying intratumoral lactic acidosis by TILA-TACE will be a potential protocol for hypervascular primary tumor in the liver and neuroendocrine tumor liver metastasis. Further prospective clinical trials are needed to verify the effectiveness of TILA-TACE.

## Conclusion

In summary, DPS is a rare paraneoplastic syndrome associated with SFTs, characterized by NICTH. We report the first case of metastatic SFTs in the liver, pancreas, and thoracic spine with DPS, which was successfully treated with TILA-TACE. This led to a clinical cure of DPS in a 3-year follow-up and subsequently earned the opportunity for tumor resection. Therefore, we suggest that TILA-TACE be recommended not only for patients with SFTs and DPS but also for patients with hypervascular primary or metastatic liver tumors when curative surgery is not applicable.

## Data availability statement

The original contributions presented in the study are included in the article/supplementary material. Further inquiries can be directed to the corresponding authors.

## Ethics statement

The study was approved by the Medical Ethics Committee of the Second Affiliated Hospital of Zhejiang University School of Medicine and was performed in accordance with the Declaration of Helsinki of 2013 for Human Research. Written informed consent for publication of the clinical details and images was obtained from the patient.

## Author contributions

XS and MC conceptualized the study. KJ analyzed and interpreted all data and wrote the manuscript. SZ and LL collected and assembled data and wrote the manuscript. JW and YW analyzed the data. LL and WC interpreted study data and revised the manuscript. WG revised the manuscript. All authors contributed to the article and approved the submitted version.

## Funding

This work was supported by the National Natural Science Foundation of China (grant number 81300083 to XS), Zhejiang Provincial Medical and Health Technology Project (grant numbers 2020380946 and 2022502078 to XS), and Major Project of Zhejiang Provincial Medical and Health (2018c03009 to MC). The funders had no role in the study design, data collection and analysis, decision to publish, or preparation of the manuscript.

## Acknowledgments

We thank Professor Xun Hu (Cancer Institute, The Second Affiliated Hospital, Zhejiang University School of Medicine) for critical reading of this manuscript and constructive comments.

## Conflict of interest

The authors declare that the research was conducted in the absence of any commercial or financial relationships that could be construed as a potential conflict of interest.

## Publisher’s note

All claims expressed in this article are solely those of the authors and do not necessarily represent those of their affiliated organizations, or those of the publisher, the editors and the reviewers. Any product that may be evaluated in this article, or claim that may be made by its manufacturer, is not guaranteed or endorsed by the publisher.

## Abbreviations

WHO, World Health Organization; SFTs, solitary fibrous tumors; DPS, Doege–Potter syndrome; NTCTH, non-islet cell tumor hypoglycemia; TACE, transcatheter arterial chemoembolization; cTACE, conventional transcatheter arterial chemoembolization; TILA-TACE, targeting-intratumoral-lactic-acidosis transcatheter-arterial-chemoembolization; IGF, insulin-like growth factors; IGFBP, insulin-like growth factor binding protein; HPF, high-power field; MRI, magnetic resonance imaging; CT, computed tomography; HbA1c, hemoglobin A1c; AFP, alpha-fetoprotein; CA19-9, carbohydrate antigen 19-9; CEA, carcinoembryonic antigen.
